# Supplementary material for: Phytochemically Derived Zingerone Nanoparticles Inhibit Cell Proliferation, Invasion and Metastasis in Human Oral Squamous Cell Carcinoma
Source: Biomedicines. 2022 Jan 29;10(2):320. doi: 10.3390/biomedicines10020320 (PMC8869513; doi:10.3390/biomedicines10020320)
Supplement: Supplementary file 1 [file biomedicines-10-00320-s001.zip › biomedicines-1520095-supplementary.pdf]

Phytochemical-derived zingerone nanoparticles fight against cell invasion and metastasis in human oral squamous cell carcinoma

Cheng-Mei Yang [a,b], Tian-Huei Chu [c], Kuo-Wang Tsai [d], Shuchen Hsieh [e] and Mei-Lang Kung [f *]

a. Department of Stomatology, Kaohsiung Veterans General Hospital, Kaohsiung, Taiwan.

b. Department of Dental Technology, Shu-Zen Junior College of Medicine and Management, Kaohsiung, Taiwan.

c. Laboratory of Medical Research, Center for Education and Faculty Development, Kaohsiung Armed Forces General Hospital, Kaohsiung 80284, Taiwan

d. Department of Research, Taipei Tzu Chi Hospital, Buddhist Tzu Chi Medical Foundation, New Taipei City, Taiwan.

e. Department of Chemistry, National Sun Yat-sen University, Kaohsiung, Taiwan

f. Department of Medical Education and Research, Kaohsiung Veterans General Hospital, Kaohsiung, Taiwan

*Corresponding Author:
Dr. Mei-Lang Kung
Department of Medical Education and Research, Kaohsiung Veterans General Hospital, Kaohsiung, Taiwan 813414
Phone: +886 7 342-2121 ext 71501
Fax: +886 7 346-8056
Email: kungmeilang@gmail.com

Supplementary information

Figure legends

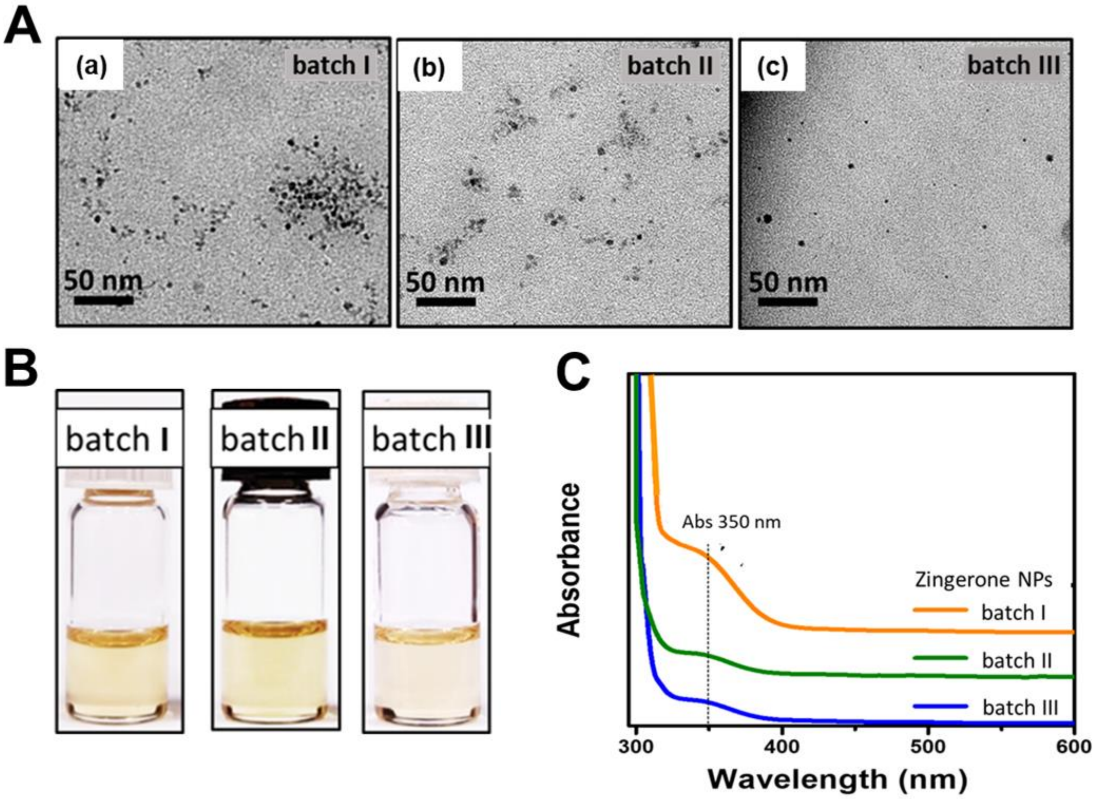

Figure S1. Characterization of zingerone NPs reproducibility. To demonstrate the zingerone NP reproducibility, three batches of zingerone NPs, which were fabricated at different times, were subjected to several experiments including TEM, photograph images, and UV absorption spectra. (A) The TEM images showed similar zingerone nanoparticle formation, and the particle sizes calculated were 1.67±0.89 nm, 2.07±1.35 nm and 1.78±1.27 nm in batch I, II and III, respectively. (B) The photographs showed the zingerone NP resulting from thre different batches with the same golden yellow color. Moreover, (**C**) the UV absorption peak of the zingerone NPs was found at 350 nm from three batches (batch I, II and III). These results indicated that zingerone NPs were highly reproducible.

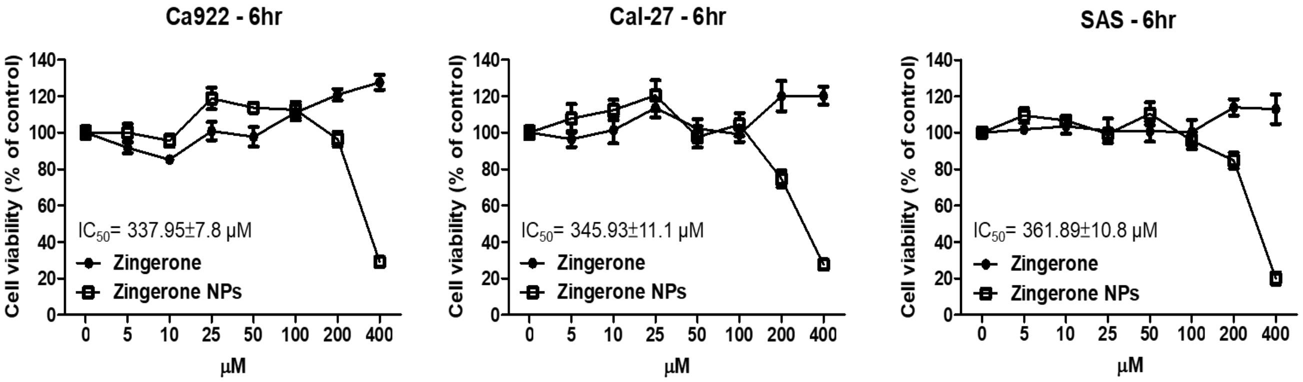

Figure S2. Cytotoxicity effects of Zingerone NPs at short-term incubation of OSCC cell lines. Cells were treated with various doses of zingerone and/or zingerone NPs for 6 h. The MTT assay was performed to analyze the cytotoxicity. This data suggested that even the zingerone NPs only induced limited cytotoxicity of these OSCC cell lines at short-term treatment. Moreover, the IC_{50} of zingerone NPs was calculated that all higher than 330 μM in these OSCC cell lines. Data are expressed as mean ± SEM of three experiments.

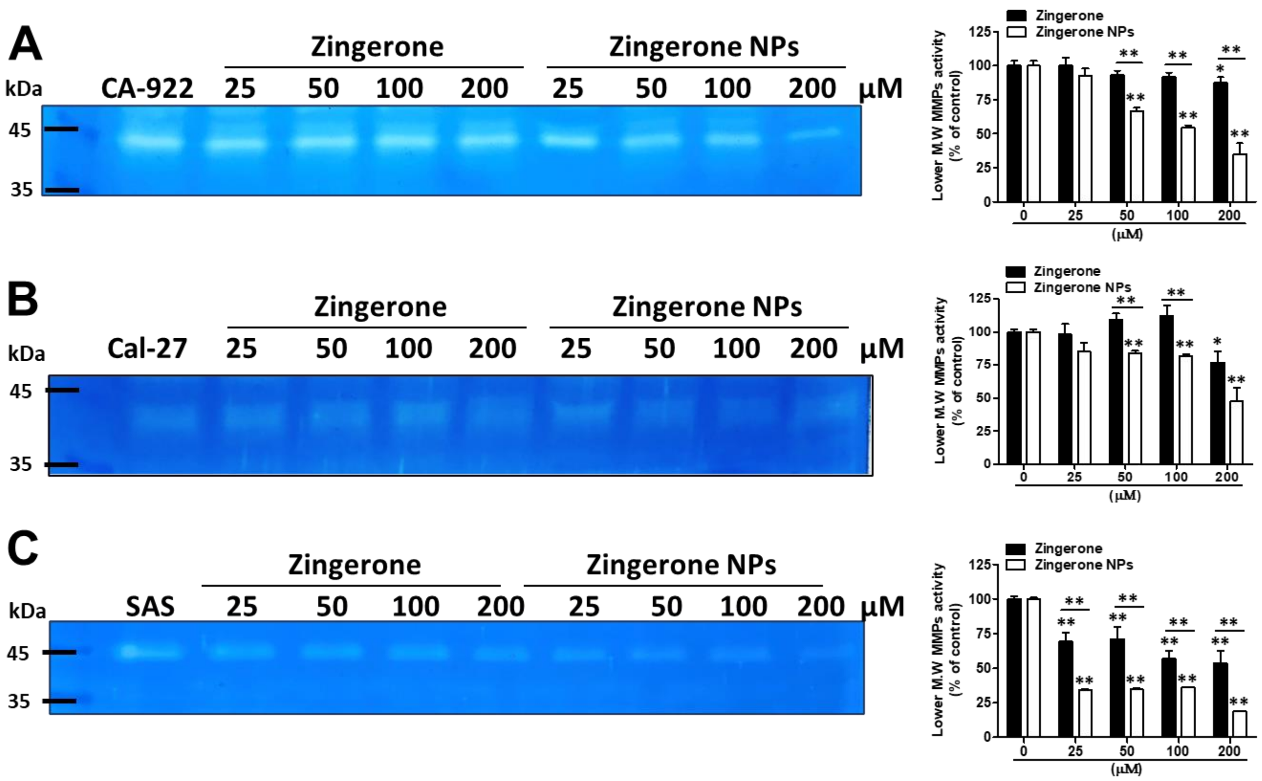

Figure S3. Effect of zingerone NPs on the enzyme activity of lower molecular weight MMPs in human OSCC cell lines. Cells were cultured in serum-free DMEM contained with various doses of zingerone and/or zingerone NPs for 24 h. The conditioned media of (A) Ca9-22 cells, (B) Cal-27 cells, and (C) SAS cells were collected to analyze MMPs activity using gelatin zymography assay. The activities of lower molecular weight MMPs (~45 kDa) in these OSCC cell lines were further analyzed and quantitated (right panels). * p<0.05, ** p<0.01.

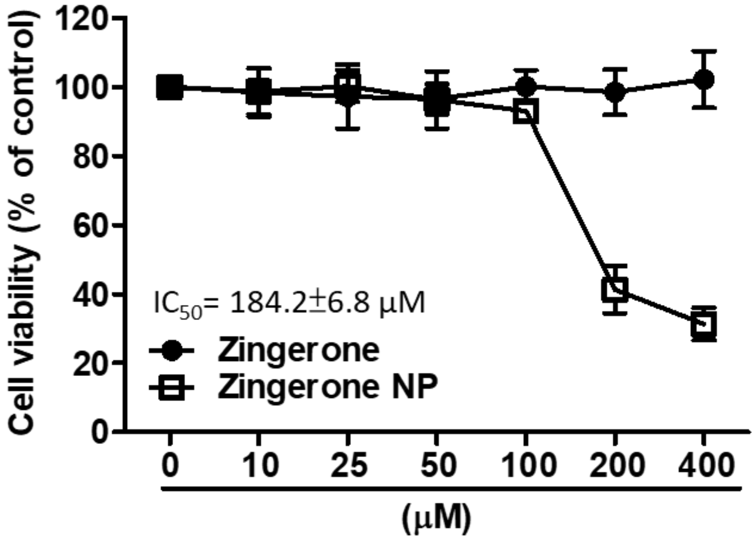

Figure S4. Effect of Zingerone NPs on cytotoxicity of normal human bone marrow HS-5 cells. Normal human HS-5 cells were purchased from ATCC (CRL-11882) and cultured in DMEM medium supplemented with 10% fetal bovine serum (Gibco). Cells were seeded in a 96-well culture plate (2×10^4 cells/well) for overnight. Cells were then treated with various doses of zingerone and/or zingerone NPs for 24 h. The effect of zingerone and/or zingerone NP on cell viability was analyzed and recorded using an MTT assay. Data are expressed as mean ± SEM of three experiments.
